# A multi-center, randomized controlled trial by the Integrative Management in Japan for Epidemic Disease (IMJEDI study-RCT) on the use of Kampo medicine, kakkonto with shosaikotokakikyosekko, in mild-to-moderate COVID-19 patients for symptomatic relief and prevention of severe stage: a structured summary of a study protocol for a randomized controlled trial

**DOI:** 10.1186/s13063-020-04746-9

**Published:** 2020-10-02

**Authors:** Shin Takayama, Takao Namiki, Takashi Ito, Ryutaro Arita, Hajime Nakae, Seiichi Kobayashi, Tetsuhiro Yoshino, Tomoaki Ishigami, Koichiro Tanaka, Mosaburo Kainuma, Kotaro Nochioka, Airi Takagi, Masaru Mimura, Takuhiro Yamaguchi, Tadashi Ishii

**Affiliations:** 1 Department of Kampo Medicine, Tohoku University Hospital, 1-1, Seiryo-machi, Aoba-ku, Sendai, 980-8574 Japan; 2 Department of Education and Support for Regional Medicine, Tohoku University Hospital, 1-1, Seiryo-machi, Aoba-ku, Sendai, 980-8574 Japan; 3 Department of Kampo and Integrative Medicine, Tohoku University Graduate School of Medicine, 1-2, Seiryo-machi, Aoba-ku, Sendai, 980-8575 Japan; 4 Department of Japanese-Oriental (Kampo) Medicine, Graduate School of Medicine, Chiba University, 1-8-1, Inohana, Chuo-ku, Chiba, 260-8670 Japan; 5 Akashi Clinic Kanda, 3-8, Kandaogawa-machi, Chiyoda-ku, Tokyo, 101-0052 Japan; 6 Department of Emergency and Critical Care Medicine, Akita University Graduate School of Medicine, 1-1-1, Hondo, Akita, 010-8543 Japan; 7 Department of Respiratory Medicine, Japanese Red Cross Ishinomaki Hospital, Nishimichishita-71, Hebita, Ishinomaki, 986-8522 Japan; 8 Center for Kampo Medicine, Keio University School of Medicine, 35, Shinanomachi, Shinjuku-ku, Tokyo, 160-8582 Japan; 9 Department of Cardiology, Yokohama City University Hospital, 3-9, Fukuura, Kanazawa-ku, Yokohama, 236-0004 Japan; 10 Department of Traditional Medicine, Faculty of Medicine, Toho University, 6-11-1, Omori-nishi, Ota-ku, Tokyo, 143-8541 Japan; 11 Community Medicine Education Unit, Graduate School of Medical Sciences, Kyushu University, 3-1-1, Maidashi, Higashi-ku, Fukuoka, 812-8582 Japan; 12 Department of Cardiovascular Medicine, Tohoku University Graduate School of Medicine, 1-1, Seiryo-machi, Aoba-ku, Sendai, Miyagi 980-8574 Japan; 13 Clinical Research Data Center, Tohoku University Hospital, 1-1, Seiryo-machi, Aoba-ku, Sendai, Miyagi 980-8574 Japan; 14 Department of Neuropsychiatry, Keio University School of Medicine, 35, Shinanomachi, Shinjuku-ku, Tokyo, 160-8582 Japan; 15 Division of Biostatistics, Tohoku University Graduate School of Medicine, 1-1, Seiryo-machi, Aoba-ku, Sendai, Miyagi 980-8574 Japan

**Keywords:** COVID-19, Randomized controlled trial, Protocol, Kampo medicines, Symptom relief, Prevention for severe stage

## Abstract

**Objectives:**

We aimed to test our hypothesis that additional administration of traditional Japanese (Kampo) medicine, kakkonto (kakkon-to: KT) and shosaikotokakikyosekko (sho-saiko-to-ka-kikyo-sekko: SSKKS), is more effective in relieving symptoms and preventing the onset of severe infection in mild-to-moderate COVID-19 patients compared to those treated only with conventional treatment.

**Trial design:**

The study is designed as a multi-center, interventional, parallel-group, randomized (1:1 ratio), investigator-sponsored, two-arm study.

**Participants:**

Patients and inpatients will be recruited from 8 Japanese academic and non-academic hospitals. The inclusion and exclusion criteria are as follows:

*Inclusion criteria*:

1. Diagnosed as positive for severe acute respiratory syndrome coronavirus 2 (SARS-CoV-2)
2. Clinical stages of mild-to-moderate COVID-19
3. Symptomatic
4. ≥ 20 years of age
5. Male or female
6. Ability to communicate in Japanese
7. Outpatients and inpatients
8. Provided informed consent

*Exclusion criteria*:

1. Difficulty in providing informed consent due to dementia, psychosis, or psychiatric symptoms
2. Allergic to Kampo or Western medicines used in this study
3. Pregnant and lactating
4. Unable to follow up
5. Participating in another clinical trial or interventional study
6. Hypokalemic or taking oral furosemide or steroids
7. Determined unsuitable for this study by the physician

**Intervention and comparator:**

Patients in the control group will receive conventional treatment with antipyretics, painkillers, or antitussives for symptoms that occurred after they contracted the SARS-CoV-2 infection.

Patients in the Kampo group will receive 2.5 g of KT (TJ-1@TSUMURA and Co.) and 2.5 g of SSKKS (TJ-109@TSUMURA and Co.) 3 times a day, orally, for 14 days in addition to the conventional treatment as mentioned above.

**Main outcomes:**

The number of days till at least one of the symptoms (fever, cough, sputum, malaise, shortness of breath) improves in the first 14 days of treatment. To assess the cough, sputum, malaise, and shortness of breath, a numeric rating scale will be used to define improvement in terms of a 2-point decrease in the number of days from the start of treatment for at least 2 days. Fever will be defined as an improvement when the temperature is less than 37 °C.

**Randomization:**

Patients are randomized (1:1 ratio) to each group using the minimization method, with balancing of the arms with severity of disease stage and patient age (< 65, 65 to < 75, or ≥ 75 years). Computer-generated random numbers will be used for the minimization method.

**Blinding (masking):**

Open-label with no blinding

**Numbers to be randomized (sample size):**

The main research hypothesis of this study is that the combination of Kampo medicine and conventional treatment will significantly improve the patients’ symptoms (fever, fatigue, cough, sputum, and shortness of breath) during the first 14 days of treatment as compared with conventional treatment alone. Concerning the analysis of the primary endpoint, the duration of time before improvement of at least one of the common cold-like symptoms (fever, malaise, cough, sputum, and shortness of breath) will be estimated using the Kaplan-Meier method, and the survival curves will be compared between groups using the log-rank test. Assuming this method of analysis and based on previous studies reporting the efficacy of Kampo medicine for COVID-19 and H1N1 influenza patients, the median survival time in the Kampo medicine group is estimated as 3 days; this time will be 1.5 times longer in the control group. Assuming a one-sided significance level of 5%, a power of 70%, and an allocation ratio of 1:1, the required sample size is calculated as 126 cases. To compensate for a loss in follow-up, we plan to include 150 cases in both groups (Kampo group = 75, control group = 75).

**Trial status:**

Protocol version 1.2 as of August 20, 2020

Recruitment start (expected): October 1, 2020

Recruitment finish (expected): October 31, 2023

**Trial registration:**

Japan Registry of Clinical Trials (jRCT) jRCTs021200020. Registered on August 25, 2020

**Full protocol:**

The full protocol is attached as an additional file and is accessible from the *Trials* website (Additional file 1). In the interest of expediting the dissemination of this material, the familiar formatting has been eliminated; this Letter serves as a summary of the key elements of the full protocol.

..

## Supplementary information

**Additional file 1.**

## Data Availability

Not applicable.

