# Supplementary material for: A multi-center, randomized controlled trial by the Integrative Management in Japan for Epidemic Disease (IMJEDI study-RCT) on the use of Kampo medicine, kakkonto with shosaikotokakikyosekko, in mild-to-moderate COVID-19 patients for symptomatic relief and prevention of severe stage: a structured summary of a study protocol for a randomized controlled trial
Source: Trials. 2020 Oct 2;21:827. doi: 10.1186/s13063-020-04746-9 (PMC7530547; doi:10.1186/s13063-020-04746-9)
Supplement: Supplementary file 1 — Additional file 1. [file 13063_2020_4746_MOESM1_ESM.pdf]

臨床研究法に規定される臨床研究

軽症、中等症 COVID-19 患者の感冒様症状に対する漢方薬追加投与に関する多施設共同ランダム化比較試験

（　登録番号　：　jRCTs021200020　）

研究代表医師

高山 真

東北大学病院　総合地域医療教育支援部　准教授

〒980-8574　宮城県仙台市青葉区星陵町 1-1

TEL/FAX：022-728-3036

E-mail : takayama@med.tohoku.ac.jp

研究事務局

高山 真

東北大学病院　総合地域医療教育支援部　准教授

〒980-8574　宮城県仙台市青葉区星陵町 1-1

TEL/FAX：022-728-3036

E-mail : takayama@med.tohoku.ac.jp

2020 年 7 月 3 日　作成（第 1.1 版）

目次

0. 概要 0
0.1. シェーマ 0
0.2. 目的 0
0.3. 対象 0
0.4. 治療 1
0.5. 予定症例数、研究期間 1
0.6. 問合せ先 1
1. 臨床研究の実施体制に関する事項 2
1.1. 研究代表医師 2
1.2. 実施医療機関および研究責任医師 2
1.3. 研究分担医師 2
1.4. 研究調整事務局 2
1.5. 研究責任医師以外の臨床研究に従事する者の氏名・役割 2
1.6. 効果安全性評価委員会の役割 4
1.7. 研究に関する問合せ窓口 4
1.8. 本研究における研究責任医師、実施医療機関の要件 4
2. 臨床研究の背景に関する事項 4
2.1. 国内外における対象疾患の状況 4
2.2. これまでに実施されてきた標準治療の経緯及び内容 5
2.3. 現在の標準治療の内容及び治療成績 5
2.4. 当該臨床研究の必要性につながる、現在の標準治療の課題、不明点等 5
2.5. 当該臨床研究に用いる医薬品等に関する情報 5
3. 臨床研究の目的に関する事項 9
4. 臨床研究の内容に関する事項 9
4.1. 臨床研究の内容、期間 9
4.1.1. 実施される臨床研究の種類及び手法 9
4.1.2. バイアスを最小限にする又は避けるために取られる無作為化及び盲検化等の方法の説明、無作為化の手順 10
4.1.3. 臨床研究に用いる医薬品等の用法・用量の説明 11
4.1.4. 臨床研究の対象者の参加期間 11
5. 臨床研究の対象者の選択及び除外並びに臨床研究の中止に関する基準 12
5.1. 研究対象者の選定方針 12
5.1.1. 適格基準 12
5.1.2. 除外基準 12
5.2. 臨床研究の一部及び全体の中止規定又は中止基準の説明 12
6. 臨床研究の対象者に対する治療 13
6.1. 治療内容 13
6.2. 検査スケジュール 13
6.3. 併用療法 13
6.4. 後治療 14
7. 有効性の評価に関する事項 14

7.1. 主要評価項目 14
7.2. 副次的評価項目 14
7.3. 評価の中央判定 14
8. 安全性の評価に関する事項 14
8.1. 有害事象の定義 14
8.2. 有害事象の収集・評価 14
8.3. 疾病等の定義 17
8.4. 疾病等発生時の必要な措置 17
8.5. 実施医療機関の管理者への疾病等報告 17
8.6. 認定臨床研究審査委員会への疾病等報告 17
8.7. 厚生労働大臣への疾病等報告 19
8.8. 効果安全性評価委員会への対応 19
8.9. 共同研究機関への報告 19
8.10. 試験薬提供者、資金提供者への報告等 19
8.11. 定期報告 19
8.11.1. 実施医療機関の管理者への定期報告 19
8.11.2. 認定臨床研究審査委員会への定期報告 20
8.11.3. 厚生労働大臣への定期報告 20
9. 統計的な解析に関する事項 21
9.1. 統計解析の方法 21
9.2. 中間解析 21
9.3. 予定症例数、設定根拠 21
9.3.1. 予定症例数 21
9.3.2. 設定根拠 22
9.3.3. 研究対象者登録見込み 22
10. 原資料等の閲覧に関する事項 22
10.1. 原資料の特定 22
10.2. 原資料の直接閲覧 22
11. 品質管理及び品質保証に関する事項 22
11.1. データの管理方法、自己点検の方法 22
11.1.1. 症例記録の作成 22
11.1.2. CRF の入力方法 22
11.2. 原資料 23
11.3. 症例報告書中の入力内容を原資料とすべき項目の特定 23
11.4. モニタリング・監査の実施体制、実施手順 23
11.4.1. モニタリング 23
11.4.2. 監査 23
11.5. 不適合の管理 24
11.5.1. 不適合 24
11.5.2. 重大な不適合 24
12. 倫理的な配慮に関する事項 24
12.1. 規制要件の遵守 24
12.2. 認定臨床研究審査委員会への申請 24
12.3. 厚生労働大臣への実施計画の届出 24
12.4. 研究の進捗状況や研究継続に関する審査・承認（定期報告） 24

12.5.　研究対象者に生じる負担、予測されるリスク（起こりうる有害事象を含む）・利益、これらの総合的評価、負担・リスクを最小化する対策 24
13.　記録（データを含む。）の取扱い及び保存に関する事項 25
13.1.　試料・情報の授受に関する記録の作成・保管 25
13.2.　試料・情報が同意を受ける時点では特定されない将来の研究のために用いられる可能性／他の研究機関に提供する可能性がある場合、その旨と同意を受ける時点において想定される内容 25
13.3.　試料・情報等の保存・廃棄の方法 25
13.3.1.　保存 25
13.3.2.　廃棄 26
14.　臨床研究の実施に係る金銭の支払及び補償に関する事項 26
14.1.　研究対象者等に経済的負担または謝礼がある場合、その旨、その内容 26
14.2.　健康被害に対する補償の有無、内容 26
15.　臨床研究に関する情報の公表に関する事項 26
16.　臨床研究の実施期間 26
17.　臨床研究の対象者に対する説明及びその同意（これらに用いる様式を含む。）に関する事項 26
17.1.　インフォームド・コンセントを受ける手続 26
17.2.　同意 27
18.　前各号に掲げるもののほか、臨床研究の適正な実施のために必要な事項 27
18.1.　研究の資金源等、研究機関の研究に係る利益相反及び個人の収益等、研究者等の研究に係る利益相反に関する状況 27
18.2.　知的財産 27
18.3.　業務内容、委託先の監督方法 27
18.4.　個人情報等の取扱い 28
18.4.1.　個人情報の利用目的 28
18.4.2.　利用方法（匿名化の方法） 28
18.4.3.　安全管理責任体制（個人情報の安全管理措置） 29
18.5.　研究対象者等、その関係者からの相談等への対応 29
18.6.　研究計画書の変更 29
18.7.　総括報告書 30
19.　引用文献 30
改定履歴（使用時は本ページを削除） 31

0. 概要

0.1. シェーマ

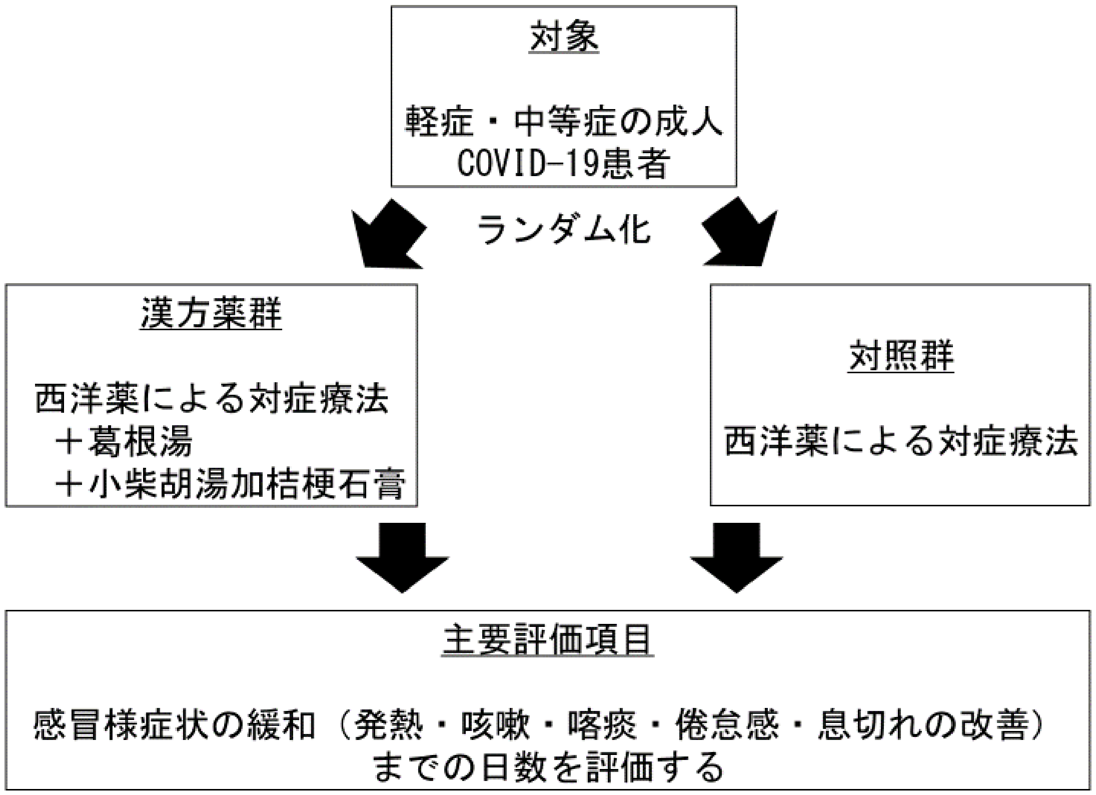

0.2. 目的

軽症、中等症 COVID-19 患者の感冒様症状に対する、一般的対症療法への漢方薬追加投与による症状改善効果を、西洋薬を用いた一般的対症療法を対照に多施設共同ランダム化比較試験にて検討する。

0.3. 対象

軽症、中等症の成人COVID-19患者

適格基準

(1) 疾患名: COVID-19
(2) 病期、ステージ: 軽症から中等症
　軽症：臨床症状が軽微で画像検査では肺炎像なし
　中等症：発熱、気道症状あり、画像上肺炎像を認めるが、呼吸や酸素化に異常なし
(3) 感冒様症状を有する
(4) 年齢：20歳以上（登録時）
(5) 性別：男性、女性
(6) 日本語で意思疎通が可能
(7) 外来、入院
(8) 研究参加について本人から文書で同意が得られる

0.4. 治療

両群において、COVID-19治療薬を含む西洋薬を用いた治療を対症療法とする。

西洋薬は、解熱鎮痛剤、鎮咳剤、去痰剤、制吐剤、整腸剤を、患者の症状に合わせて、通常用量にて使用する。本研究は保険診療で行う多施設共同研究のため、各施設の採用薬状況を優先し、薬剤は制限しない。また、本疾患治療に使用される COVID-19 治療薬も含まれ、使用の制限は無い。

・対照群：対症療法として、COVID-19治療薬を含む西洋薬を投与

・漢方薬併用群：対症療法に加えて、ツムラ葛根湯7.5g/日＋ツムラ小柴胡湯加桔梗石膏7.5g/日、分3毎食前投与、14日間追加投与

0.5. 予定症例数、研究期間

(1) 予定症例数：150例

(2) 研究期間：2020年6月（jRCT公開後）～2024年12月

（登録期間：～2023年10月、追跡期間：～2023年12月）

0.6. 問合せ先

(1) 適格基準、治療変更基準等、臨床的判断を要するもの：

高山 真　東北大学病院　総合地域医療教育支援部

〒980-8574　宮城県仙台市青葉区星陵町1-1　TEL/FAX: 022-728-3036

(2) 登録手順、記録用紙（CRF）記入等：

高山 真　東北大学病院　総合地域医療教育支援部

〒980-8574　宮城県仙台市青葉区星陵町1-1　TEL/FAX: 022-728-3036

東北大学病院臨床試験データセンター

高木愛理・特任助教

〒980-8574　宮城県仙台市青葉区星陵町 1-12　TEL: 022-717-7137

1. 臨床研究の実施体制に関する事項

1.1. 研究代表医師

高山 真　東北大学病院　総合地域医療教育支援部　准教授
〒980-8574　宮城県仙台市青葉区星陵町1-1　TEL/FAX: 022-728-3036
E-mail：takayama@med.tohoku.ac.jp

1.2. 実施医療機関および研究責任医師

実施医療機関	職名・氏名
東北大学病院　総合地域医療教育支援部<br>〒980-8574　宮城県仙台市青葉区星陵町 1-1　TEL: 022-728-3036<br>E-mail：takayama@med.tohoku.ac.jp	准教授<br>高山 真
別紙：実施医療機関リスト参照	

1.3. 研究分担医師

研究分担医師：「研究分担医師リスト」参照

1.4. 研究調整事務局

研究事務局(医局)
高山真、准教授、東北大学病院　総合地域医療教育支援部
〒980-8574　宮城県仙台市青葉区星陵町1-1　TEL/FAX: 022-728-3036
小野理恵、医員、同上
有田龍太郎、医員、同上

調整事務局
シミックヘルスケア・インスティテュート株式会社
SSIカンパニー　エリア長（東北エリア担当）
新井　直美
〒980-8485 宮城県仙台市青葉区中央1-2-3仙台マークワン　14F
TEL　022-216-6061　　Mobile 070-6995-0368　　FAX　022-216-6066
E-mail　naomi-arai@cmicgroup.com

1.5. 研究責任医師以外の臨床研究に従事する者の氏名・役割

(1) データマネジメント責任者
東北大学病院臨床試験データセンター
山口拓洋・教授
〒980-8574 宮城県仙台市青葉区星陵町 1-1
TEL:022-717-7659, FAX:022-717-7580

(2) モニタリング責任者

東北大学病院臨床試験データセンター
川邉庸介・助手
〒980-8574 宮城県仙台市青葉区星陵町 1-1
TEL:022-717-7659, FAX:022-717-7580

(3) 監査責任者
東北大学病院　臨床試験品質保証室
早坂　幸子・助手
〒980-8574　宮城県仙台市青葉区星陵町１番１号
Tel：022-717- 8752

(4) 統計解析担当者
東北大学病院臨床試験データセンター
高木愛理・特任助教
〒980-8574　宮城県仙台市青葉区星陵町 1-12
TEL: 022-717-7137

(5) 研究・開発計画支援担当者
東北大学病院臨床研究推進センター
プロトコル作成支援部門
後岡 広太郎・部門長（特任准教授）
〒980-8574 宮城県仙台市青葉区星陵町１番１号
TEL：022-717-7122（代表）　FAX：022-717-7104

(6) 研究協力者（プロトコル作成支援・CRC）
・プロトコル作成支援
東北大学病院臨床研究推進センター
プロトコル作成支援部門
草場　美津江（特任准教授）
佐藤　真由美（助手）
星　絢子（特任助教）
〒980-8574 宮城県仙台市青葉区星陵町１番１号
TEL：022-717-7122（代表）　FAX：022-717-7104
・CRC
東北大学病院　総合地域医療教育支援部・漢方内科
桑原　安輝子・技術補佐員
〒980-8574　宮城県仙台市青葉区星陵町 1-1
Tel/FAX：022-728-3036

(7) 割付責任者
東北大学病院臨床試験データセンター
高木愛理・特任助教
〒980-8574　宮城県仙台市青葉区星陵町 1-1
TEL: 022-717-7137　FAX：022-717-7580

1.6. 効果安全性評価委員会の役割

委員長
菅野　武
東北大学病院　総合地域医療教育支援部
〒980-8574　宮城県仙台市青葉区星陵町 1-1
Tel/FAX：022-717-7587

大田　英揮
東北大学病院　放射線科
〒980-8574　仙台市青葉区星陵町 1－1
TEL 022-717-7312

院外
片寄　大
利府掖済会病院
〒981-0103 宮城県宮城郡利府町森郷字新太子堂 51 番地
Tel.022-767-2151　Fax.022-767-2156

1.7. 研究に関する問合せ窓口

(1) 研究対象者（研究参加者）の登録方法：
高山 真
東北大学病院　総合地域医療教育支援部　准教授
〒980-8574　宮城県仙台市青葉区星陵町 1-1
TEL/FAX：022-728-3036
E-mail：takayama@med.tohoku.ac.jp

(2) 有害事象発生時の対応方法：
高山 真
東北大学病院　総合地域医療教育支援部　准教授
〒980-8574　宮城県仙台市青葉区星陵町 1-1
TEL/FAX：022-728-3036
E-mail：takayama@med.tohoku.ac.jp

1.8. 本研究における研究責任医師、実施医療機関の要件

研究代表医師は研究開始前に、本研究の参加施設へ要件を満たしていることを確認する。
研究開始後に参加施設を新たに追加する場合も、新規申請時と同様とする。

2. 臨床研究の背景に関する事項

2.1. 国内外における対象疾患の状況

新型コロナウイルス Severe acute respiratory syndrome coronavirus 2 (SARS-Cov-2) は 2019 年 11 月に中国湖北省武漢から発生が確認され、WHO は 2020 年 1 月 30 日公衆衛生上の非常事態宣言（public

health emergency of international concern）を出したが 2020 年 3 月時点で世界中に拡大し猛威を振るっている。コロナウイルスは元来かぜの病原ウイルスとして発見されたが、Severe acute respiratory syndrome coronavirus, Middle East respiratory syndrome coronavirus などの病原性の高いウイルスが過去にも出現してきた。SARS-Cov-2 の特徴として、無症状病原体保持者がいること、潜伏期間が 1-14 日（中央値 5 日間）と長いこと、現在唯一の検査法である PCR 法も感度が低いこと、患者は 4 週ほど PCR 検査陽性が続き感染を拡大させる可能性があること、治療法が確立していないこと、特に高齢者や合併症をもつ患者では重症化し死に至ることがあること、が挙げられる。抗マラリア薬、抗ウイルス薬等を試験的に使用した症例報告もあるが、十分なエビデンスはまだ得られていない。

2.2. これまでに実施されてきた標準治療の経緯及び内容

本疾患における確立された標準治療は無く、症状に対する対症療法のみである。

2.3. 現在の標準治療の内容及び治療成績

現在日本の方針では、PCR 陽性でも軽症例では対症療法のみで経過観察する方針となっているとなる。しかしながらそうした軽症例から重症化する症例もあり、軽症例への適切な治療も極めて重要と考えられる。中国では軽症例に対して中医（日本でいう漢方医学）による治療を行っている。中国政府は新型コロナウイルス感染症(COVID-19)診療方案を公表しており、その中にも中医治療が記載されている（文献 1）。また、西洋薬と中薬を併用した観察研究では確定診断例の治療には清肺排毒湯が用いられ、肺炎が改善した症例報告がされているほか、98 例に 9 日間の清肺排毒湯治療を行った観察研究では主症状に有効率が 91.6%であったと報告している（文献 2，3）。清肺排毒湯は生薬を煎じた煎じ薬であり日本では使用しない生薬も含まれていてそのまま実現するのは難しいが、日本で保険適応のある漢方エキス製剤の葛根湯と小柴胡湯加桔梗石膏を組み合わせることでも一部を再現できる。これらは通常多くのウイルス性上気道感染症（かぜ症候群）の症状である発熱、咳、痰、悪心嘔吐に用いられる漢方薬であり、入手は容易である。また、この組み合わせは柴葛解肌湯という処方名で、大正時代のスペイン風邪流行の時に広く用いられてほとんど死者を出さなかったという記録が残されている。こうした経緯をふまえた多くの漢方専門家の議論の結果、葛根湯と小柴胡湯加桔梗石膏を組み合わせた処方を COVID-19 に対する治療薬の候補とした。

有効な治療法がないとされる中で日本の伝統医学である漢方医学の効果を検討することは、今後の COVID-19 の拡大を想定する中で極めて重要と考える。

2.4. 当該臨床研究の必要性につながる、現在の標準治療の課題、不明点等

現在のところ、抗ウイルス薬、抗 HIV 薬、免疫抑制薬などの治験が進行中であるが、明らかな有効性を示す報告はないことから、軽症から中等症における治療は対症療法のみであり、症状のみならず重症化を抑制する薬剤の開発、解明が喫緊の課題である。

2.5. 当該臨床研究に用いる医薬品等に関する情報

① 試験治療（試験薬）の作用機序、特徴を基に、試験治療を選択した根拠を記載する。

・ツムラ葛根湯エキス顆粒（医療用）（以下葛根湯）

構成生薬：麻黄は気管支拡張作用、鎮咳作用。葛根は鎮痙作用、桂皮は解熱、鎮痛作用、芍薬は鎮痙鎮痛作用、甘草は抗炎症作用。生姜、大棗は健胃作用。全体で漢方の初期に広く使用することから試験薬として設定。

・ツムラ小柴胡湯加桔梗石膏エキス顆粒（医療用）（以下小柴胡湯加桔梗石膏）

構成生薬：柴胡は抗炎症作用。人参は強壮作用。半夏は鎮咳作用。甘草は抗炎症作用。黄芩は抗炎症作用。生姜、大棗は健胃作用。桔梗は鎮咳去痰作用。石膏は抗炎症作用。全体で消化管、内臓の抗炎症作用と健胃作用があることから試験薬に設定。

② 試験治療の対象疾患での適応承認の有無、承認用法・用量を記載する。

・ツムラ葛根湯エキス顆粒（医療用）

【効能又は効果】

自然発汗がなく頭痛、発熱、悪寒、肩こり等を伴う比較的体力のあるものの次の諸症： 感冒、鼻かぜ、熱性疾患の初期、炎症性疾患（結膜炎、角膜炎、中耳炎、扁桃腺炎、乳腺炎、リンパ腺炎）、肩こり、上半身の神経痛、じんましん

【用法及び用量】

通常、成人１日7.5gを2～3回に分割し、食前又は食間に経口投与する。なお、年齢、体重、症状により適宜増減する。

【使用上の注意】

1. 慎重投与（次の患者には慎重に投与すること）

(1) 病後の衰弱期、著しく体力の衰えている患者［副作用があらわれやすくなり、その症状が増強されるおそれがある。］

(2) 著しく胃腸の虚弱な患者［食欲不振、胃部不快感、悪心、嘔吐等があらわれることがある。］

(3) 食欲不振、悪心、嘔吐のある患者［これらの症状が悪化するおそれがある。］

(4) 発汗傾向の著しい患者［発汗過多、全身脱力感等があらわれることがある。］

(5) 狭心症、心筋梗塞等の循環器系の障害のある患者、又はその既往歴のある患者

(6) 重症高血圧症の患者

(7) 高度の腎障害のある患者

(8) 排尿障害のある患者

(9) 甲状腺機能亢進症の患者

2. 重要な基本的注意

(1) 本剤の使用にあたっては、患者の証（体質・症状）を考慮して投与すること。なお、経過を十分に観察し、症状・所見の改善が認められない場合には、継続投与を避けること。

(2) 本剤にはカンゾウが含まれているので、血清カリウム値や血圧値等に十分留意し、異常が認められた場合には投与を中止すること。

(3) 他の漢方製剤等を併用する場合は、含有生薬の重複に注意すること。

3. 相互作用

薬剤名等	臨床症状・措置方法	機序・危険因子
(1)マオウ含有製剤<br>(2)エフェドリン類含有製剤<br>(3)モノアミン酸化酵素（MAO）阻害剤<br>(4)甲状腺製剤<br>チロキシン<br>リオチロニン<br>(5)カテコールアミン製剤<br>※※アドレナリン<br>イソプレナリン<br>(6)キサンチン系製剤<br>テオフィリン<br>ジプロフィリン	不眠、発汗過多、頻脈、動悸、全身脱力感、精神興奮等があらわれやすくなるので、減量するなど慎重に投与すること。	交感神経刺激作用が増強されることが考えられる。
(1)カンゾウ含有製剤<br>(2)グリチルリチン酸及びその塩類を含有する製剤	偽アルドステロン症があらわれやすくなる。また、低カリウム血症の結果として、ミオパチーがあらわれやすくなる。（「重大な副作用」の項参照）	グリチルリチン酸は尿細管でのカリウム排泄促進作用があるため、血清カリウム値の低下が促進されることが考えられる。

4. 副作用

本剤は使用成績調査等の副作用発現頻度が明確となる調査を実施していないため、発現頻度は不明である。

（1）重大な副作用

1）偽アルドステロン症：低カリウム血症、血圧上昇、ナトリウム・体液の貯留、浮腫、体重増加等の偽アルドステロン症があらわれることがあるので、観察（血清カリウム値の測定等）を十分に行い、異常が認められた場合には投与を中止し、カリウム剤の投与等の適切な処置を行うこと。

2）ミオパチー：低カリウム血症の結果としてミオパチーがあらわれることがあるので、観察を十分に行い、脱力感、四肢痙攣・麻痺等の異常が認められた場合には投与を中止し、カリウム剤の投与等の適切な処置を行うこと。

3）肝機能障害、黄疸：AST（GOT）、ALT（GPT）、Al-P、γ-GTP の上昇等を伴う肝機能障害、黄疸があらわれることがある ので、観察を十分に行い、異常が認められた場合には投与を中止し、適切な処置を行うこと。

（2）その他の副作用

5. 高齢者への投与一般に高齢者では生理機能が低下しているので減量するなど注意すること。

6. 妊婦、産婦、授乳婦等への投与妊娠中の投与に関する安全性は確立していないので、妊婦又は妊娠している可能性のある婦人には、治療上の有益性が危険性を上回ると判断される場合にのみ投与すること。

7. 小児等への投与　小児等に対する安全性は確立していない。[使用経験が少ない]

8. その他の注意　湿疹、皮膚炎等が悪化することがある。

【薬効薬理】

1．抗アレルギー作用

羊赤血球誘発遅延型足蹠浮腫反応（SRBC-DTH）マウスに経口前投与したところ、浮腫が抑制された。

2．インフルエンザウィルス感染症に対する作用

経口前投与したマウスにインフルエンザウィルスを感染させたところ、発熱が抑制され、死亡率が低下した。

3．作用機序

本剤は、以下の作用により薬理効果を示すことが示唆されている。

(1) プロスタグランジンE2（PGE2）に対する作用

・ウサギ培養アストロサイトにおいて、ブラジキニンによるPGE2生成を、短時間処理（10min.）では抑制し、長時間処理（18hr）では増加させた。また、長時間処理（18hr）で内因性のPGE2遊離を抑制した（in vitro）。

・C6ラットグリオーマ細胞において、カルシウムイオノフォアA23187によるPGE2遊離促進を抑制した（in vitro）。

(2) サイトカインに対する作用

経口前投与したマウスにインフルエンザウィルスを感染させたところ、肺胞洗浄液中及び血清中のインターロイキン（IL）-1α濃度の上昇が抑制された 2)。また、肺胞洗浄液中のIL-12 濃度が上昇した 5)。

・ツムラ小柴胡湯加桔梗石膏エキス顆粒（医療用）

【効能又は効果】

咽喉がはれて痛む次の諸症： 扁桃炎、扁桃周囲炎

【用法及び用量】

通常、成人 1 日 7.5g を 2～3 回に分割し、食前又は食間に経口投与する。なお、年齢、体重、症状により適宜増減する。

【使用上の注意】

1. 慎重投与（次の患者には慎重に投与すること）
   (1) 胃腸の虚弱な患者［食欲不振、胃部不快感、軟便、下痢等があらわれることがある。］
   (2) 著しく体力の衰えている患者［副作用があらわれやすくなり、その症状が増強されるおそれがある。］
2. 重要な基本的注意
   (1) 本剤の使用にあたっては、患者の証（体質・症状）を考慮して投与すること。なお、経過を十分に観察し、症状・所見の改善が認められない場合には、継続投与を避けること。
   (2) 本剤にはカンゾウが含まれているので、血清カリウム値や血圧値等に十分留意し、異常が認められた場合には投与を中止すること。
   (3) 他の漢方製剤等を併用する場合は、含有生薬の重複に注意すること。

薬剤名等	臨床症状・措置方法	機序・危険因子
(1)カンゾウ含有製剤<br>(2)グリチルリチン酸及びその塩類を含有する製剤	偽アルドステロン症があらわれやすくなる。また、低カリウム血症の結果として、ミオパチーがあらわれやすくなる。（「重大な副作用」の項参照）	グリチルリチン酸は尿細管でのカリウム排泄促進作用があるため、血清カリウム値の低下が促進されることが考えられる。

3. 相互作用併用注意（併用に注意すること）
4. 副作用

本剤は使用成績調査等の副作用発現頻度が明確となる調査を実施していないため、発現頻度は不明である。

（1）重大な副作用

1）偽アルドステロン症：低カリウム血症、血圧上昇、ナトリウム・体液の貯留、浮腫、体重増加等の偽アルドステロン症があらわれることがあるので、観察（血清カリウム値の測定等）を十分に行い、異常が認められた場合には投与を中止し、カリウム剤の投与等の適切な処置を行うこと。

2）ミオパチー：低カリウム血症の結果としてミオパチーがあらわれることがあるので、観察を十分に行い、脱力感、四　肢痙攣・麻痺等の異常が認められた場合には投与を中止し、カリウム剤の投与等の適切な処置を行うこと。 3）肝機能障害、黄疸：AST（GOT）、ALT（GPT）、Al-P、γ-GTP の上昇等を伴う肝機能障害、黄疸があらわれることがあるので、観察を十分に行い、異常が認められた場合には投与　を中止し、適切な処置を行うこと。

（2）その他の副作用

5. 高齢者への投与一般に高齢者では生理機能が低下しているので減量するなど注意すること。
6. 妊婦、産婦、授乳婦等への投与妊娠中の投与に関する安全性は確立していないので、妊婦又は妊娠している可能性のある婦人には、治療上の有益性が危険性を上回ると判断される場合にのみ投与すること。
7. 小児等への投与

小児等に対する安全性は確立していない。［使用経験が少ない］

8. その他の注意　類似処方の小柴胡湯では、間質性肺炎、膀胱炎の副作用が報告されている。特に、間質性肺炎はインターフェロン-α との併用例で多く報告されている。

3. 臨床研究の目的に関する事項

軽症、中等症 COVID-19 患者に対する一般的対症療法への漢方薬追加投与による感冒様症状緩和効果を、西洋薬を用いた一般的対症療法を対照に多施設共同ランダム化比較試験にて検討する。

主要評価項目は外来受診後 14 日間における感冒様症状の緩和（解熱（37℃未満)、咳嗽改善、喀痰改善、倦怠感改善、息切れ改善）までの日数を評価する。咳嗽、喀痰、倦怠感、息切れの評価には NRS を用い２ポイント低下した状態が２日以上継続した場合を改善とする。副次的評価項目は、外来受診後 14 日目までの重症化（SpO2≤93%, 呼吸数≥30/分, 酸素投与）の有無について検討する。

4. 臨床研究の内容に関する事項

4.1. 臨床研究の内容、期間

4.1.1. 実施される臨床研究の種類及び手法

⑴研究デザイン

①対照：西洋薬による一般的対症療法
②盲検化：オープンラベル
③ランダム化：ランダム化
④比較方法：並行群間比較
⑤群数：2 群

⑵研究デザインの設定根拠

優越性を示すための比較試験

⑶研究の手順

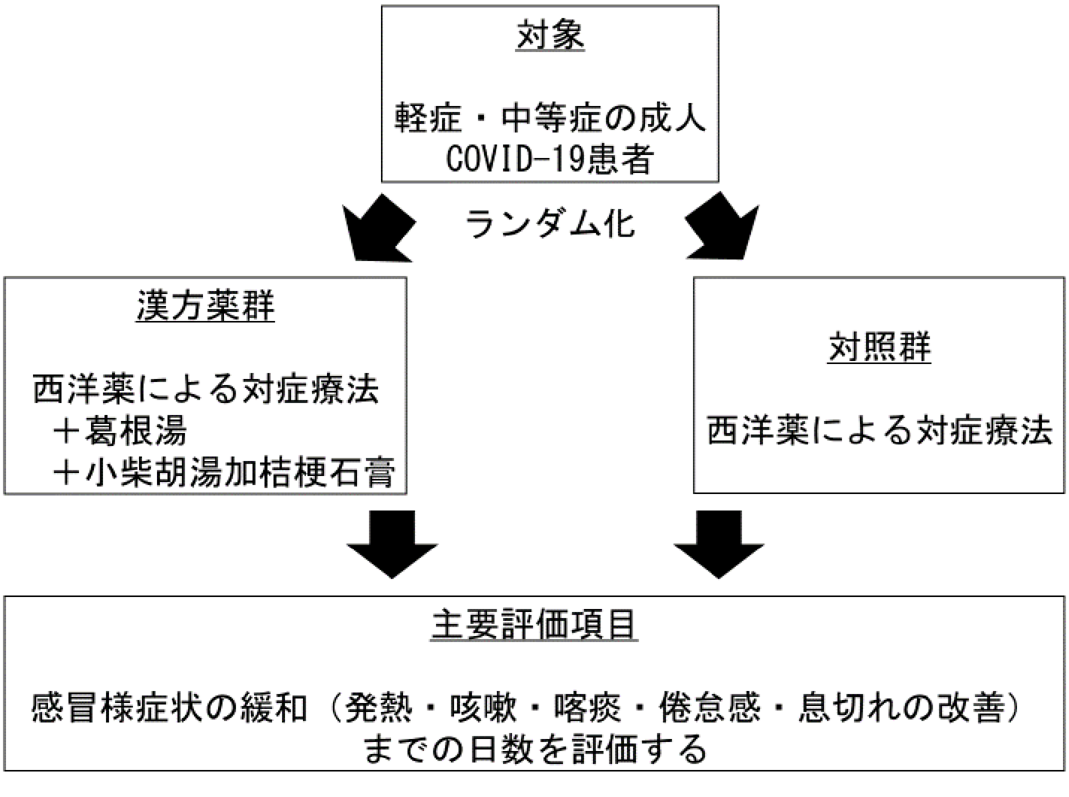

・外来受診もしくは入院にて、病歴聴取、診察、検査を一般診療と同様行う。
・本研究への参加を説明し、書面で同意を取得しカルテ記載後に症例登録を行う。
・症例登録はEDCシステム（Viedoc）によるWeb登録とし、適格基準を満たした研究対象者に対して、本登録を行う。研究対象者は、別途作成する手順や割付仕様に基づき設定された同システム上で各群に割付けられる。
・有効性評価項目：主要評価項目は感冒様症状の緩和（解熱（37℃未満）、咳嗽改善、喀痰改善、倦怠感改善、息切れ改善）までの日数、副次的評価項目は重症化転帰（SpO2≤93%, 呼吸数≥30/分, 酸素投与）である。
・安全性評価項目は、新たな手足のしびれ・浮腫（偽アルドステロン症の確認目的）、皮疹等（アレルギー症状の確認目的）、胃部不快感等（消化器症状の確認目的）の新たな症状の出現である。
・「登録前」　時期：外来受診時もしくは入院時、検査項目：低カリウム血症確認のための採血、重症度確認のための胸部レントゲン写真、COVID-19確定診断のためのPCR検査等
・「治療中」　時期：投薬開始から7日目
・「治療終了後」　時期：
検査項目：投薬開始から7日目、症状・投薬状況・有害事象・内服状況の確認。投薬開始から14日後に症状・投薬状況・有害事象・重症化・死亡に関する確認。
・外来受診後14日目に症状、転帰の確認を行うが、確認するタイミングの3日の遅れは許容される。
・研究終了もしくは中止となった後の治療制限は設けない。

・西洋薬による治療について
西洋薬は解熱鎮痛剤、鎮咳剤、去痰剤、制吐剤、整腸剤を、患者の症状に合わせて、通常用量にて使用する。本研究は保険診療で行う多施設共同研究のため、各施設の採用薬状況を優先し、薬剤を制限しない。また、本疾患治療に使用される COVID-19 治療薬も含まれ、使用の制限は無い。

例）
・解熱鎮痛剤：アセトアミノフェン
・鎮咳薬：
チペピジンヒベンズ酸塩（アスベリン）、
デキストロメトルファン臭化水素酸塩水和物（メジコン）、
ジモルファンリン酸塩（アストミン）、
エプラジノン塩酸塩（レスプレン）、コデイン製剤
・去痰薬：
カルボシステイン（ムコダイン）、
アンブロキソール塩酸塩（ムコソルバン）、
フドステイン（クリアナール）
・制吐剤：
メトクロプラミド（プリンペラン）、
ナウゼリン（ドンペリドン）
・整腸剤：活性生菌製剤（ミヤ BM、ビオフェルミン、ビオスリー、レベニン）
・COVID-19 治療薬

4.1.2.　バイアスを最小限にする又は避けるために取られる無作為化及び盲検化等の方法の説明、

無作為化の手順

(1)登録の手順

感冒症状がありCOVID-19が疑われる症例に、通常診療、PCR検査等を行う。本研究の説明を行った後、書面同意を得て登録、カルテにその旨を記載する。通常は書面で同意書を取得し原本を保管するが、本疾患は感染性の高い伝染病であり患者との接触時間や患者が接触したものは感染性があると判断される。これらを鑑み本研究ではやむを得ず原本は患者本人が持ちその写真を事務局で保管する。

中央割り付けによるランダム化の後、西洋薬治療群あるいは漢方薬併用群に沿った治療を行う。

誤登録・重複登録が判明した場合、速やかに研究事務局に連絡する。

(2)無作為化手順、盲検化手順、割付調整因子

以下を割付因子とした動的割り付けを行う。

1. 重症度：軽症、中等症

軽症：臨床症状が軽微で画像検査では肺炎像なし

中等症：発熱、気道症状あり、画像上肺炎像を認めるが、呼吸や酸素化に異常なし

2. 65歳未満、65歳以上75歳未満、75歳以上

割付の詳細な手順は割付手順書等に記載し、割付責任者が保管する。

(3)割付調整因子設定の根拠

既報により、重篤化する割合が異なるため。合併症は年齢と交絡するため。

4.1.3.　臨床研究に用いる医薬品等の用法・用量の説明

(1) 医薬品等

・葛根湯
・小柴胡湯加桔梗石膏

(2) 用法・用量

・葛根湯
用法及び用量は1日7.5gを3回に分割し、食前又は食間に14日間経口投与する。
・小柴胡湯加桔梗石膏
用法及び用量は1日7.5gを3回に分割し、食前又は食間に14日間経口投与する。

14日間の投薬の間で症状が改善し、内服の必要性が無くなった際には内服量を減量し、葛根湯・小柴胡湯加桔梗石膏　各5g 朝・夕食前内服に減量して14日目まで内服は継続する。漢方薬内服で胃もたれなど胃腸症状の増悪があった際には、食前もしくは食間内服を食後内服に変更する。延期や休止は行わない。

(3) プラセボ及び対照薬を含む臨床研究に用いる医薬品等の管理の手順

本研究に用いる医薬品は保険適応で処方される。各共同研究施設より処方され、連携する院外、院内薬局保管、管理される。

4.1.4.　臨床研究の対象者の参加期間

各研究対象者の参加期間は、同意取得日から試験薬投与期間の最終観察日又は中止検査時のいずれか遅い時点までとする。各研究対象者の研究参加予定期間は2週間である。

5. 臨床研究の対象者の選択及び除外並びに臨床研究の中止に関する基準

5.1. 研究対象者の選定方針

5.1.1. 適格基準

(1) 疾患名: COVID-19
(2) 病期、ステージ: 軽症から中等症
軽症：臨床症状が軽微で画像検査では肺炎像なし
中等症：発熱、気道症状あり、画像上肺炎像（すりガラス陰影や浸潤影など）を認めるが、呼吸や酸素化に異常なし
(3) 感冒様症状を有する（咳嗽、喀痰、倦怠感、息切れの症状で、いずれかが「NRSで2ポイント以上」）
(4) 年齢：20歳以上（登録時）
(5) 性別：男性、女性
(6) 日本語で意思疎通が可能
(7) 外来、入院
(8) 研究参加について本人から文書で同意が得られる

5.1.2. 除外基準

(1) 認知症、精神病または精神症状などで本人からの同意取得が困難
(2) 本研究で用いる漢方薬、西洋薬に対するアレルギーを有する患者
(3) 妊娠中、授乳中の患者
(4) フォローアップができない可能性のある患者
(5) 治験、その他の介入研究に参加中の患者
(6) 低カリウム血症、フロセミド内服中、ステロイド内服中の患者
(7) 担当医師が本研究の対象として好ましくないと判断した方

5.2. 臨床研究の一部及び全体の中止規定又は中止基準の説明

漢方薬によるアレルギー反応（皮疹など）が重篤な場合、消化器症状が増悪し経口摂取が困難な場合、原疾患の増悪の際には中止とする。

・増悪／再発：原病の増悪・再発による治療中止。無効を含めて増悪／再発／無効としてもよい。
・有害事象：担当医判断または中止規定に従った有害事象による治療中止。
・拒否（有害事象）：有害事象に関連する研究対象者拒否による治療中止。
・拒否（その他）：転居による場合等、有害事象に関連しない研究対象者拒否による治療中止。
・死亡：プロトコル治療中の死亡（因果関係の有無を問わない）。

その他、研究の継続が困難と判断された場合、研究責任医師は、効果・安全性評価委員会に「研究早期中止許可願い」を提出する。提出された内容に基づき、効果・安全性評価委員会より研究早期中止勧告が出された場合には、研究早期中止の手続きに入る。

6. 臨床研究の対象者に対する治療

6.1. 治療内容

両群における一般的対症療法については全ての医薬品についての明記は困難である。使用される対症療法薬剤についてもランダム化されて施設内での取り決めにより使用されることから、両群同等となると考えられる。また、入院、通院、食事についても同様の考え方に基づく。

漢方薬治療

・葛根湯

用法及び用量は 1 日 7.5g を 3 回に分割し、食前又は食間に 14 日間経口投与する。

・小柴胡湯加桔梗石膏

用法及び用量は 1 日 7.5g を 3 回に分割し、食前又は食間に 14 日間経口投与する。

6.2. 検査スケジュール

	day 1					Day7	day 14-17
	体温、血圧、脈拍、レントゲン、採血、新型コロナウイルス検査（PCR 等）	同意取得	登録	ランダム化	処方	健康管理・症状チェックシートの確認、投薬状況の確認、有害事象の確認	受診から 14 日目 (3 日の延長可)
症状 (咳嗽、喀痰、倦怠感、息切れ)	○						○
投薬状況確認						○	○
酸素投与の有無							○
死亡の有無							○
有害事象確認						○	○

検査内容、問診内容は「通常診療の範囲内」で行われる。

「転帰確認」の方法は外来診察、入院診察、電話により症状経過確認により行われる。

評価項目の各症状については、健康管理・症状チェックシートを対象者に配布して、「咳嗽、喀痰、倦怠感、息切れ」はすべて自己申告（自覚症状）転帰確認の際に「日数」についてのデータを得る。

6.3. 併用療法

⑴許容する併用療法

・本疾患発症以前から使用中の薬剤（例：降圧薬や抗血小板剤、経口血糖降下剤など）

・咳嗽が激しい場合には、西洋薬の鎮咳剤の追加は許容される。

・抗菌薬の日常診療に基づいた通常治療は許容される。

⑵許容されない併用療法

・感冒、気管支炎、胃腸炎に対する漢方薬の追加併用。

6.4. 後治療

14日以降の後治療については各共同研究施設で行われる診療で対応する。

7. 有効性の評価に関する事項

7.1. 主要評価項目

治療開始後14日間の感冒様症状（発熱、咳嗽、喀痰、倦怠感、息切れ）のうち少なくとも1つが改善するまでの日数
尚、咳嗽、喀痰、倦怠感、息切れの評価については、NRSを用い、治療開始時よりも2ポイント低下した日数が2日以上継続した場合を改善と定義する。発熱は37℃未満を改善とする。

7.2. 副次的評価項目

治療開始後14日間の重症化の有無
治療開始後14日間における、感冒様症状（発熱、咳嗽、喀痰、倦怠感、息切れ）の全ての項目が改善するまでの日数、およびそれぞれの項目が改善するまでの日数重症化の有無に寄与する因子の検討（発熱、咳嗽、喀痰、倦怠感、息切れ、群、時点、割付因子など）
安全性評価項目は、手足のしびれ、浮腫、皮疹等のアレルギー症状、胃部不快感（電話で医師、医療スタッフが確認）。

7.3. 評価の中央判定

評価の中央判定を行わない。

8. 安全性の評価に関する事項

8.1. 有害事象の定義

有害事象とは、実施された研究との因果関係の有無を問わず、研究対象者に生じた全ての好ましくない又は意図しない傷病若しくはその徴候（臨床検査値の異常を含む）をいう。

8.2. 有害事象の収集・評価

研究者等は、発現したすべての有害事象に関し、有害事象名、程度（重篤、非重篤）、重篤と判断した理由、発現日、転帰日、処置、転帰（回復、軽快、回復したが後遺症あり、未回復、死亡）、試験薬との因果関係、コメント（因果関係と判定理由等）を症例報告書に記載する。
有害事象の収集は、研究への参加以降、個々の研究対象者の観察期間終了または中止時までの期間とする。

①有害事象名は、原則として診断名・疾患名（病名）で症例報告書に記載する。診断名・疾患名が特定できない場合や研究者等が診断名・疾患名としないことが妥当と判断した場合、臨床症状または徴候（臨床検査値の異常を含む）を有害事象名として症例報告書に記載する。
②有害事象を治療のために研究対象者に対して取られた処置（あり／なし）を記載する。
③試験薬に対して取られた措置を記載する。
・なし（試験薬の使用を中止しなかった場合）

・使用休止（試験薬の使用を一旦中止し、その後使用を再開した場合）

・使用中止（試験薬の使用を中止した場合）

④有害事象の転帰を記載する。

転帰の分類	解説
**回復**	有害事象が消失、または元の状態まで戻っている
**軽快**	有害事象は完全に回復していないものの、ほぼ消失、またはほぼ元の状態に戻っている
**回復したが後遺症あり**	有害事象は元の状態まで回復したものの、後遺症が残っている
**未回復**	有害事象は継続中である
**死亡**	有害事象の結果、死亡した

⑤試験薬との因果関係を記載する。

因果関係	判定基準
**関連あり**	・リチャレンジ陽性（再投与による再発）<br>・因果関係が確立されており明らか<br>・発現までの時間に説得力がある<br>・デチャレンジ陽性（投与中止で消失）<br>・交絡するリスク因子がない<br>・曝露量や曝露期間との整合性がある<br>・正確な既往歴による裏付けがある<br>・その症例の場合明らかで容易に評価できる<br>・併用治療が原因である可能性が低い<br>・他に説明できる原因がない<br>・その他、担当医師による判断
**関連なし**	・試験薬使用との因果関係を証明できる因子がない

CIOMS VI Working Group Report,

Management of Safety Information from Clinical Trials, Appendix 7, 2005

⑥対象疾患、標準治療、試験治療の内容等により、予測される有害事象の許容範囲を設定する。治療関連死亡が予測される場合、過去の研究での頻度を示し、許容範囲と設定根拠を記載する（幅のある記載も可）。許容範囲は参考値であり、統計学的な記載は不要。

⑦研究計画書治療および研究計画書で規定された検査で用いる薬剤で予期される有害反応は、薬剤添付文書の最新版を参照のこと。

薬剤添付文書は、独立行政法人医薬品医療機器総合機構の検索ページより入手できる。

医療用医薬品 情報検索ページ http://www.pmda.go.jp/PmdaSearch/iyakuSearch/

⑧がんの臨床試験では多くの場合、死亡まで追跡することから、多くの「原疾患（がん）による有害事象」が多数観察されることになり、追跡期間中の有害事象データをすべて一律に収集することは現実的でないため、有害事象の定義について検討する。

[1)]重症度分類

有害事象/有害反応の評価には「有害事象共通用語規準 v5.0 日本語訳 JCOG 版（NCI-CommonTerminology Criteria for Adverse Events v5.0（CTCAE v5.0）の日本語訳）」（以下、CTCAE v5.0-JCOG）を用いる。なお、CTCAE v5.0-JCOG のうち、臨床検査値の施設基準値で Grade が定義されている項目については、個々の医療機関における施設基準値の代わりに「JCOG 共用基準範囲」を用いる。「JCOG 共用基準範囲」の詳細は JCOG ウェブサイト

（http://www.jcog.jp/doctor/tool/kijun.html）を参照する。

NCI CTCAE 分類に該当する項目がない場合、以下**「有害事象の重症度分類基準」**を参考に判定する。

重症度分類<br>（NCI CTCAE Grade）	基準
**軽症 （Grade1）**	症状がない、または軽度の症状がある。臨床所見または検査所見のみ。<br>治療を要さない。
**中等症 （Grade2）**	最小限/局所的/非侵襲的治療を要する。<br>年齢相応の身の回り以外の日常生活動作の制限*。
**重症 （Grade3）**	重症または医学的に重要であるが、ただちに生命を脅かすものではない。<br>入院または入院期間の延長を要する。<br>活動不能/動作不能。身の回りの日常生活動作の制限**。
**最重症 （Grade4）**	生命を脅かす。緊急の処置を要する。
**死亡 （Grade5）**	有害事象による死亡。

*身の回り以外の日常生活動作（instrumental ADL）

：食事の準備、日用品や衣類の買い物、電話の使用、金銭の管理等。

**COVID―19による入院は除く。

**身の回りの日常生活動作（self care ADL）

：入浴、着衣・脱衣、食事の摂取、トイレの使用、薬の服用が可能で、寝たきりではない状態。

[2]重篤の定義

①死に至るもの
②生命を脅かすもの
③治療のための入院又は入院期間の延長が必要となるもの
④永続的又は顕著な障害・機能不全に陥るもの
⑤子孫に先天異常を来すもの

研究計画書で規定する入院、研究前（同意取得前）より予定していた療法または検査を研究実施中に実施することのみを目的とした入院（予定手術や検査等）、有害事象に伴う治療・検査の目的以外の入院（健康診断等）は重篤な有害事象として取扱わない。

研究者等（担当医）は、重篤な有害事象／不具合が発現した場合、適切な処置を行い、研究機関の研究責任医師に報告する。

[3]予測性の定義

〇予測できない（未知）

当該事象等の発現、あるいは発現数、発現頻度、発現条件等の発現傾向が当該試験薬／試験機器に関する公式文書（添付文書）から予測できないもの

〇予測できる（既知）

当該事象等の発現、あるいは発現数、発現頻度、発現条件等の発現傾向が当該試験薬／試験機器に関する公式文書（同上）から予測できるもの

8.3. 疾病等の定義

疾病等とは、臨床研究の実施に起因するものと疑われる疾病、障害若しくは死亡または感染症に加え、臨床検査値の異常や諸症状を含む。

8.4. 疾病等発生時の必要な措置

重篤な有害事象、非重篤な有害事象は投与開始日を基点として有害事象を収集・記録する。登録日に存在する臨床所見は基礎疾患とし、有害事象とはしない。ただし、基礎疾患の増悪は有害事象として記録する。原則として、研究との因果関係有りと判断された疾病等、及び重篤な有害事象を可能な限り回復または軽快まで追跡する。ただし、死亡・障害・後遺症など、それ以上の回復が困難または追跡不能であると研究責任医師が判断した時点で追跡を終了とする。研究との因果関係の無い非重篤な有害事象は、転帰に関わらず、研究においては特に追跡を行わない。
有害事象の重篤性に関わらず、発現した有害事象は、観察期間終了日まで収集・記録する。

	重篤	非重篤
因果関係あり	回復又は軽快まで追跡	回復又は軽快まで追跡
因果関係なし	回復又は軽快まで追跡	研究においては追跡しない

8.5. 実施医療機関の管理者への疾病等報告

研究責任医師は、疾病等の発生を知った場合、下表「認定臨床研究審査委員会への疾病等の報告対象と報告期限」に記載された期限内に、その旨を当該医療機関の規定に従い当該医療機関の管理者に報告する。

多施設共同研究の場合は、加えて研究代表医師に通知する。他の参加医療機関の研究責任医師は、研究代表医師より認定臨床研究審査委員会に報告を行った旨の連絡を受けたら、当該医療機関の規定に従い当該医療機関の管理者に報告する。

8.6. 認定臨床研究審査委員会への疾病等報告

研究責任医師は、疾病等（不具合を含む）の発生を知った場合、以下の期限内に認定臨床研究審査委員会に報告する。多施設共同研究を実施する場合は研究代表医師が行う。

疾病等の発生の要因等が明らかではない場合でも、期間内にそれまでに判明している範囲で第１報として報告を行う。この場合、その後速やかに詳細な要因等について続報として報告を行い、続報は必ずしも定める期間内でなくてもよい。

報告を受けた認定臨床研究審査委員会が研究責任医師に対し意見を述べた場合、研究責任医師は、意見を尊重して必要な措置をとる。

認定臨床研究審査委員会への疾病等の報告対象と報告期限

研究分類	報告対象	報告期限
（ｉ）未承認･適応外医薬品等を用いる特定臨床研究	ア　以下の疾病等の発生のうち、臨床研究の実施によるものと疑われるものであって予測できないもの<br>①　死亡<br>②　死亡につながるおそれのある疾病等	７日

	イ　以下の疾病等の発生のうち、臨床研究の実施によるものと疑われるもの（アを除く）<br>①　死亡<br>②　死亡につながるおそれのある疾病等	15日
	ウ　以下の疾病等の発生のうち、臨床研究の実施によるものと疑われるものであって予測できないもの（アを除く）<br>①　治療のために医療機関への入院または入院期間の延長が必要とされる疾病等<br>②　障害<br>③　障害につながるおそれのある疾病等<br>④　①から③並びに死亡及び死亡につながるおそれのある疾病等に準じて重篤である疾病等<br>⑤　後世代における先天性の疾病または異常	15日
**（ⅱ）（ⅰ）以外の特定臨床研究**	ア　死亡（感染症*1によるものを除く）の発生のうち、臨床研究の実施によるものと疑われるもの	15日
	イ　以下の疾病等（感染症*1を除く）の発生のうち、臨床研究の実施によるものと疑われるものであって、かつ、臨床研究に用いた医薬品等の添付文書または容器若しくは被包に記載された使用上の注意（使用上の注意等）から予測することができないものまたは医薬品等の使用上の注意等から予測することができるものであって、その発生傾向を予測することができないもの若しくはその発生傾向の変化が保健衛生上の危害の発生若しくは拡大のおそれを示すもの<br>①　治療のために医療機関への入院または入院期間の延長が必要とされる疾病等<br>②　障害<br>③　死亡または障害につながるおそれのある疾病等<br>④　死亡または①から③の疾病等に準じて重篤である疾病等<br>⑤　後世代における先天性の疾病または異常	15日
	ウ　臨床研究の実施によるものと疑われる感染症*1による疾病等の発生のうち、医薬品等の使用上の注意等から予測することができないもの	15日
	エ　臨床研究の実施によるものと疑われる感染症*1による死亡またはイ①から⑤の疾病等の発生（ウを除く）	15日
	オ　イ①から⑤の疾病等のうち、臨床研究の実施によるものと疑われるもの（イを除く）	30日
**（ⅲ）臨床研究の実施に起因するものと疑われる疾病等の発生<br>：（ⅰ）（ⅱ）のすべてを除いたもの**		定期報告時*2

*1 感染症

生物由来製品の生物由来原料／材料から、当該医薬品等への病原体の混入が疑われる場合等。

HBV、HCV、HIV等のウイルスマーカーの陽性化も感染症報告対象となる。

*2 定期報告時

認定臨床研究審査委員会への定期報告時

8.7. 厚生労働大臣への疾病等報告

研究責任医師は、疾病等の発生を知った場合、以下の期限内に、厚生労働大臣に報告する。多施設共同研究を実施する場合は、研究代表医師が行う。

なお、厚生労働大臣への報告に際しては、医薬品の疾病等報告については、jRCTの疾病等報告サービスを活用する。

8.8. 効果安全性評価委員会への対応

(1) 効果安全性評価委員会を設置した研究の場合、研究責任医師は、以下について効果安全性評価委員会に審査を依頼する。多施設共同研究を実施する場合は、研究代表医師が行う。

①情報の評価<br>②計画変更の要否<br>③研究継続の可否<br>①　その他（新規登録の中断、説明同意文書の改訂、他の研究対象者への再同意　等）

8.9. 共同研究機関への報告

(1) 多施設共同研究の場合、研究代表医師は、有害事象が発現した研究機関の研究責任医師、共同研究機関の研究責任医師に以下を報告し、各研究機関の長への報告を依頼する。

①重篤な有害事象に関する報告書<br>②効果安全性評価委員会の審査結果

(2) 研究機関の研究責任医師は、研究機関の長の指示を受け、必要な措置を講じる。

8.10.試験薬提供者、資金提供者への報告等

(1) 研究責任医師は、試験薬提供者、資金提供者に、以下を報告する。

①（参考書式３）重篤な有害事象に関する報告書<br>②効果安全性評価委員会の審査結果

(2) 研究期間中、研究責任医師は、試験薬／試験機器の安全性に関する新たな情報（緊急安全性情報、研究報告、製品回収等の措置報告等）を調査する。適宜、試験薬／試験機器提供者、資金提供者からこれらの情報を入手する。

(3) 研究の継続に影響を及ぼすと考えられる情報を入手した場合、研究責任医師は効果安全性評価委員会への審査依頼等、必要な措置を講じる。

(4) 多施設共同研究を実施する場合、上記(1)~(3)については、研究代表医師が行う。

8.11.定期報告

8.11.1. 実施医療機関の管理者への定期報告

研究責任医師は、定期的に臨床研究の実施状況について、下表「認定臨床研究審査委員会への定期報告事項と報告期限」に記載された期限内に、その旨を当該医療機関の規定に従い当該医療機関の管理者に報告する。多施設共同研究を実施する場合は、研究代表医師が自身の所属する医療機関の管理者に報告する。

多施設共同研究の場合は、他の参加医療機関の研究責任医師は、研究代表医師より認定臨床研究審査委員会に報告を行った旨の連絡を受けたら、当該医療機関の規定に従い当該医療機関の管理者に報

告する。

8.11.2. 認定臨床研究審査委員会への定期報告

研究責任医師は、定期的に臨床研究の実施状況について、以下の期限内に、認定臨床研究審査委員会に報告する。多施設共同研究を実施する場合、研究代表医師が行う。

報告を受けた認定臨床研究審査委員会が研究責任医師に対し意見を述べた場合、研究責任医師は、意見を尊重して必要な措置をとる。

認定臨床研究審査委員会への定期報告事項と報告期限

報告事項	報告期限
① 参加した対象者数*1<br>② 疾病等の発生状況、その後の経過*2<br>③ 規則、研究計画書に対する不適合の発生状況、その後の対応<br>④ 安全性・科学的妥当性についての評価*3<br>⑤ 医薬品等製造販売業者等の関与に関する事項*4	実施計画を厚生労働大臣に提出した日から起算して、１年ごとに、期間満了後2月以内（原則として）

*1 参加した対象者数

予定症例数、同意取得症例数、実施症例数、完了症例数、中止症例数、補償を行った件数

*2 疾病等の発生状況、その後の経過

既に報告・審査されているものも含め、研究全体の疾病等の発生状況の要約（簡潔に記載）

*3 安全性・科学的妥当性についての評価

疾病等の発生状況・その後の経過、不適合事案の発生状況・その後の対応等を含む臨床研究の実施状況、当該期間中に発表された研究報告等における臨床研究に用いる医薬品等に関連する有効／無効の情報を踏まえ、臨床研究の安全性・科学的妥当性の評価を記載する。

*4 医薬品等製造販売業者等の関与に関する事項

当該研究責任医師が実施する臨床研究に従事する者（研究責任医師・研究分担医師、統計的解析責任者）、研究計画書に記載されている者で当該研究を実施することによって利益を得ることが明白な者は、報告を行う時点における関与に関する事項を再度確認し、利益相反管理基準・利益相反管理計画を提出する。

確認の結果、利益相反管理基準・利益相反管理計画に変更がない場合、その旨を認定臨床研究審査委員会に報告する。

経過措置が適用された臨床研究について初めて報告する場合、関与に関する事項についての利益相反管理基準及び関与に関する事項についての利益相反管理計画を含む。

なお、実施状況に係る②~⑤の事項については、別紙（形式は問わない）の添付も差し支えない。

8.11.3. 厚生労働大臣への定期報告

① 研究責任医師は、定期的に臨床研究の実施状況について、以下の期限内に、厚生労働大臣に報告する。多施設共同研究を実施する場合、研究代表医師が行う。
② jRCTへの記録により報告したものとみなす。
③ 厚生労働大臣への報告概要は公表される。

厚生労働大臣への定期報告事項と報告期限

報告事項	報告期限[*1]
① 認定臨床研究審査委員会の名称<br>② 認定臨床研究審査委員会による臨床研究の継続の適否<br>③ 参加した対象者数	認定臨床研究審査委員会が意見を述べた日から起算して、<br>１月以内

[*1] 報告期限

国際共同研究の場合、他国と定期報告の時期を合わせるため、認定臨床研究審査委員会が認めた場合に限り、実施計画を厚生労働大臣に提出した１年以内の他国の起算日を起算日とすることを可とする。その際、初回報告は、実施計画提出日から当該起算日までの内容を取りまとめて報告する。

9. 統計的な解析に関する事項

9.1. 統計解析の方法

本試験の主たる研究仮説は治療開始後14日間における感冒様症状が対照群と比較し漢方薬併用群において有意に改善することである。主要評価項目である治療開始後14日間の感冒様症状（発熱、咳嗽、喀痰、倦怠感、息切れ）のうち少なくとも1つが改善するまでの日数を群間で比較検討するため、群ごとにカプラン・マイヤー法による生存曲線の推定を行い、改善までの期間中央値の点推定値および信頼区間を算出すると共に、ログランク検定を用いて治療群間の比較を行う。また、Cox回帰モデルを用いてハザード比の推定を行う。有意水準は片側5%とする。

尚、咳嗽、喀痰、倦怠感、息切れの評価については、NRSを用い、治療開始時よりも2ポイント低下した日数が2日以上継続した場合を改善と定義する。発熱は37℃未満を改善とする。

副次評価項目の解析は、治療開始後14日間の重症化の有無において、漢方薬併用群での重症化割合が対照群における重症化割合よりも有意に下回った場合、漢方薬併用により重症化が抑制できたと判断する。各群の治療開始後14日間の重症化割合の点推定値および群間差の点推定値、それらの信頼区間を算出し群間で比較する。

また、感冒様症状（発熱、咳嗽、喀痰、倦怠感、息切れ）の全てが改善するまで、およびそれぞれの項目が改善するまでの日数についても主要評価項目と同様に解析する。

さらに、重症化の有無を結果変数、各項目のNRSスコア、群、時点、および割付因子を説明変数とした一般化線形モデルを用いた解析を行い、重症化に寄与する因子を検討する。

安全性の解析は、治療開始から14日間の観察期間中における手手足のしびれ、浮腫、皮疹等のアレルギー症状、胃部不快感、発現件数、例数、および発現割合を群別に集計する。

9.2. 中間解析

中間解析は行わない。

9.3. 予定症例数、設定根拠

9.3.1. 予定症例数

実質予定症例数：計150例（漢方薬併用群：75例　対照群：75例）

9.3.2. 設定根拠

本試験の主たる研究仮説は、治療開始後 14 日間の感冒様症状（発熱、倦怠感・咳嗽・喀痰・息切れ）ついて、漢方薬併用群が対照群よりも有意に改善した場合、漢方薬併用により感冒様症状が緩和できたと判断する。主要評価項目では、漢方薬併用群と対象群における治療開始 14 日間の感冒様症状（発熱、倦怠感・咳嗽・喀痰・息切れ）のうち 1 つ以上が改善するまでの日数について群ごとにカプラン・マイヤー法による生存曲線の推定を行い、ログランク検定を用いて群間で比較する。この解析方法を想定し、COVID-19 および新型インフルエンザへの漢方治療による症状改善効果を検討した先行研究に基づき、対照群では漢方薬併用群と比較して､改善までに 1.2~1.5 倍長く要すると仮定し､症例数設計を行った。有意水準片側 2.5%および 5%、検出力 70%および 80%、割付比を 1:1 と仮定したところ、必要なサンプルサイズは下記と計算された。

片側有意水準 2.5%、検出力 80%の際に、両群で 210-1000 例
片側有意水準 2.5%、検出力 70%の際に、両群で 164-786 例
片側有意水準 5%、検出力 80%の際に、両群で 164-788 例
片側有意水準 5%、検出力 70%の際に、両群で 126-600 例
症例の集積可能性の観点を鑑み、離脱率 20%と仮定し、両群で 150 例を目標症例数とした。

9.3.3. 研究対象者登録見込み

海外では 1 日の発症数が 10000 例を超えている（2020 年 3 月 28 日現在)。中国の例では、感染制御に半年程を要していることから、本研究においても登録期間を半年とした。海外と同様の経過をたどるとなると、早期に予定症例数に達する見込みである。

10. 原資料等の閲覧に関する事項

10.1. 原資料の特定

対象者に対する医薬品等の投与及び診療により得られた臨床所見、観察その他の活動に関する元の記録（健康管理・症状チェックシート）やデータ（診療記録、検査記録、投与記録）等

10.2. 原資料の直接閲覧

研究責任医師及び実施医療機関は、臨床研究に関連するモニタリング、認定臨床研究審査委員会・規制当局の調査の際に、原資料等の全ての臨床研究関連記録を直接閲覧に供する。

11. 品質管理及び品質保証に関する事項

11.1. データの管理方法、自己点検の方法

11.1.1. 症例記録の作成

本試験では、EDCシステム（Viedoc）を使用する。なお、症例記録は、登録から最終評価日までの所定の評価・観察日ごとに入力し、各期間の評価終了後3日以内を目安に入力する。

11.1.2. CRF の入力方法

(1) 症例記録の入力内容および訂正に関しては研究責任医師が責任を負う。各被験者の各観察・検査が終了後、3 日以内を目安に EDC に入力する。入力方法に関しては、「EDC 入力の手引き（仮)」に

従う。

(2) 研究協力者は、原資料が存在しその客観性が保証できる場合は、原資料から症例記録に転記することが出来る。

(3) 研究責任医師は、作成された症例記録についてその内容を点検し、確認した上で電子署名する。

(4) 研究責任医師は作成した症例記録を定められた手順にて提出する。

(5) 研究責任医師は、症例記録と原資料に矛盾がある場合、その理由を説明する記録を作成の上定められた手順にて提出する。

(6) 研究責任医師または研究分担医師が症例記録を訂正する場合、「EDC 入力の手引き」に従う。

(7) 症例報告書に記載されたデータのうち、「11.3 症例報告書中の入力内容を原資料とすべき項目の特定」にある項目については、治験責任医師または評価者以外の治験分担医師が症例報告書へ直接入力する場合は、原資料の有無は不問とする。

(8) 本治験では EDC システムを使用するため、症例記録の写しは最終的にデータセンターより実施医療機関に配布する。

11.2. 原資料

原資料とは、被験者に対する試験薬の投与および、診療により得られたデータその他の記録をいう。

・ 被験者、代諾者の同意および情報提供に関する記録
・ 診療録（ワークシート、検査伝票等を含む）、看護記録など症例記録作成の基となったすべての記録
・ 試験薬の使用に関する記録

11.3. 症例報告書中の入力内容を原資料とすべき項目の特定

症例記録に記載されたデータのうち以下に示す項目は、症例記録の記載内容をもって原資料とする。

・既往歴の有無
・中止の有無、理由、コメント
・有害事象の有無、重篤性、処置の有無、転帰日、転帰

11.4. モニタリング・監査の実施体制、実施手順

11.4.1. モニタリング

研究責任医師が指名するモニタリング担当者は、品質マネジメント計画での方針と、「モニタリングに関する手順書」および「モニタリング計画書」に従い、試験実施計画書に記載されている内容ならびに臨床研究法に従って試験が実施されているかを確認するため中央モニタリングを含めた実施医療機関へのモニタリングを定期的に行う。なお、モニタリングの頻度と対象はリスクマネジメント結果に応じて変更しうる。

11.4.2. 監査

研究責任医師は、研究の科学的・倫理的な質の向上と教育を目的とする監査を監査責任者に依頼する。監査従事者は、監査手順書を作成し、監査手順書に基づき、研究機関を訪問して、研究実施機関の管理者による承認文書の確認、説明・同意文書の確認、症例報告書内容と診療録の照合等の監査を実施する。

監査結果は、作成した監査報告書を研究責任医師、実施医療機関の管理者に提出する。

11.5. 不適合の管理

11.5.1. 不適合

臨床研究法における不適合とは「臨床研究が臨床研究法施行規則または研究計画書に適合していない状態」を指す。研究責任医師は、臨床研究が不適合であると知った場合には、実施医療機関の管理者に報告するとともに、研究代表医師に通知する。

11.5.2. 重大な不適合

臨床研究の対象者の人権や安全性及び研究の進捗や結果の信頼性に影響を及ぼす場合、重大な不適合として取扱う。重大な不適合に相当する可能性がある場合、研究代表医師は状況を把握次第速やかに認定臨床研究審査委員会に報告する。

12. 倫理的な配慮に関する事項

12.1. 規制要件の遵守

本研究に関係するすべての研究者は「ヘルシンキ宣言」（日本医師会訳）、「臨床研究法」（平成 29 年法律第 16 号）2)「臨床研究法施行規則」（平成 30 年厚生労働省令第 17 号）と関連通知に従って本研究を実施する。

12.2. 認定臨床研究審査委員会への申請

本研究の実施について、認定臨床研究審査委員会の承認、各医療機関の管理者の研究実施許可を得る。

12.3. 厚生労働大臣への実施計画の届出

研究開始に先立ち、厚生労働大臣に実施計画を提出する。

12.4. 研究の進捗状況や研究継続に関する審査・承認（定期報告）

研究代表医師は、当該研究の実施状況について、研究の進捗状況や有害事象の発生状況、利益相反管理等に関する定期報告を、自らの所属する医療機関の管理者に報告した上で、認定臨床研究審査委員会に報告する。

認定臨床研究審査委員会に報告を行ったときは、研究代表医師はその旨を、速やかに他の参加医療機関の研究責任医師に情報提供する。情報を受けた研究責任医師は、速やかに、当該情報提供の内容を所属する医療機関の管理者に報告する。

12.5. 研究対象者に生じる負担、予測されるリスク（起こりうる有害事象を含む）・利益、これらの総合的評価、負担・リスクを最小化する対策

(1) 予測される利益

本研究の試験薬は、本研究の対象となる症状に対して適応が承認され保険適用されている。本研究と日常診療を比較して、研究対象者が本研究に参加することで得られる特別な診療上の利益は考えにくい。一方でCOVID-19への治療効果を新たに明らかにできる可能性はあり、この点は社会的利益が見

込める。

⑵ 予測される危険と不利益

本研究において実施する一般的対症療法は通常の保険診療として行われるものである。日常診療に比して生じる不利益は、試験薬追加による内服量と内服回数の増加がある。薬物相互作用の可能性については否定できない。

研究に参加することにより増大するリスク・不利益は、漢方薬内服による有害事象反応である胃腸障害や低カリウム血症等がある。

これらの有害事象のリスクや不利益を最小化するために、研究対象者選択基準、治療変更基準、併用療法等を慎重に検討している。また、有害事象が予測された範囲内かモニターするとともに、重篤な有害事象や予測されない有害事象が発現した場合、必要な対策を講じる。

13. 記録（データを含む。）の取扱い及び保存に関する事項

13.1. 試料・情報の授受に関する記録の作成・保管

（１）提供先の機関

高山 真
東北大学病院　総合地域医療教育支援部　准教授
〒980-8574　宮城県仙台市青葉区星陵町 1-1
TEL/FAX: 022-728-3036

（２）提供元の機関

なし

（３）提供する試料・情報の項目

情報：病歴、治療歴、副作用等発生状況、検査結果データ等

（４）提供する試料・情報の取得の経緯

本研究で利用することについて本人からインフォームド・コンセントを得たうえで取得される

（５）提供する試料・情報の提供方法

□電子的配信（e-mail,web等)

13.2. 試料・情報が同意を受ける時点では特定されない将来の研究のために用いられる可能性／他の研究機関に提供する可能性がある場合、その旨と同意を受ける時点において想定される内容

該当せず。

13.3. 試料・情報等の保存・廃棄の方法

13.3.1. 保存

保存する試料・情報等	保存期間
○研究に用いられる研究対象者情報（診療情報、検査デー	研究終了日から5年

タ、症例報告書等）<br>○試料・情報の提供に関する記録、対応表<br>○研究記録、手順書等	

13.3.2. 廃棄

研究責任医師は、人体から取得した試料・情報等を廃棄する場合、匿名化する。

14. 臨床研究の実施に係る金銭の支払及び補償に関する事項

14.1. 研究対象者等に経済的負担または謝礼がある場合、その旨、その内容

診療に伴う初診料、検査費用、試験薬等は保険診療での自己負担があるので、参加した研究対象者には研究協力費として5,000円のクオカードを提供する。

14.2. 健康被害に対する補償の有無、内容

研究対象者に健康被害が生じた場合、保険診療の範囲内で適切な治療を行い、医療費の自己負担分は研究対象者の負担とする。本研究では健康被害に対する補償を行うため、臨床研究保険に加入する。本研究との因果関係が否定できない健康被害が生じた場合、健康被害の程度と臨床研究保険の契約内容に応じて補償を行う。ただし、研究参加に過失がある場合は対象とはならない。

15. 臨床研究に関する情報の公表に関する事項

研究代表医師は、本研究の概要、進捗状況、主な結果は jRCT（https://jrct.niph.go.jp/）で公開する。ただし、研究対象者等の人権、研究者等の関係者の人権、知的財産保護のため非公開とする事項、個人情報保護の観点から研究に著しく支障が生じるため認定臨床研究審査委員会の意見を受け研究機関の長が許可した事項は非公開とする。

研究代表医師は、研究終了後、研究対象者の個人情報保護に措置を講じた上で、遅滞なく研究結果を医学雑誌等に公表する。結果の最終公表を行った場合、遅滞なく各研究機関の長に報告する。

16. 臨床研究の実施期間

2020 年 6 月（jRCT 公開後）～2024 年 12 月

17. 臨床研究の対象者に対する説明及びその同意（これらに用いる様式を含む。）に関する事項

17.1. インフォームド・コンセントを受ける手続

研究者等は、登録前に実施医療機関の承認を得た説明文書を研究対象者に渡し、以下の内容を説明する。　「研究への参加の継続について臨床研究の対象者の意思に影響を与える可能性のある情報が得られたときは、速やかに説明文書を改訂する。」

（説明文書記載事項）

①実施する臨床研究の名称、臨床研究の実施について実施医療機関の管理者の承認を受けている旨及び厚生労働大臣に実施計画を提出している旨

②実施医療機関の名称並びに研究責任医師の氏名及び職名

③対象者として選定された理由

④予期される利益及び不利益
※不利益のうち副作用等の種類が多い場合、別紙可
⑤臨床研究への参加を拒否することは任意である旨
⑥同意の撤回に関する事項
⑦臨床研究への参加を拒否することまたは同意を撤回することにより不利益な取扱いを受けない旨
⑧臨床研究に関する情報公開の方法
⑨対象者またはその代諾者（対象者等）の求めに応じて、研究計画書その他の臨床研究の実施に関する資料を入手または閲覧できる旨及びその入手または閲覧の方法
⑩対象者の個人情報の保護に関する事項
⑪試料等の保管及び廃棄の方法
⑫医薬品等製造販売業者等の関与に関する状況
⑬苦情及び問合せへの対応に関する体制
⑭臨床研究の実施に係る費用に関する事項
⑮他の治療法の有無及び内容並びに他の治療法により予期される利益及び不利益との比較
⑯臨床研究の実施による健康被害に対する補償及び医療の提供に関する事項
⑰臨床研究の審査意見業務を行う認定臨床研究審査委員会における審査事項その他臨床研究に係る認定臨床研究審査委員会に関する事項
⑱その他臨床研究の実施に関し必要な事項

17.2. 同意

研究についての説明を行い、十分に考える時間を与え、研究対象者が研究の内容をよく理解したことを確認した上で、研究への参加について依頼する。
同意説明文書を用いて書面同意を取得し、それを画像などにして電子ファイルで保存する。

18. 前各号に掲げるもののほか、臨床研究の適正な実施のために必要な事項

18.1. 研究の資金源等、研究機関の研究に係る利益相反及び個人の収益等、研究者等の研究に係る利益相反に関する状況

本研究は、国立研究開発法人日本医療研究 開発機構委託研究費（研究代表医師：石井正 教授、研究課題名「COVID-19 に対する中国 伝統薬を応用した日本人への治療薬開発と統 合治療の開発研究」）及び㈱ツムラとの共同研 究契約に基づき受け入れた研究費を使用し、㈱ツムラが製造する試験薬葛根湯、小柴胡湯加桔梗石膏の効果の検討を目的に実施する。研究代表医師である高山真准教授は株式会社ツムラとの共同研究講座を兼任している。
※兼任：東北大学に雇用された教員であり、当該講座の所属を兼ねている。

18.2. 知的財産

本研究により得られた結果やデータ、知的財産権は、東北大学に帰属する。具体的な取扱いや配分は協議して決定する。研究代表医師の知的財産の帰属先を個人とするか研究機関とするかは、所属研究機関の取り決めに従う。

18.3. 業務内容、委託先の監督方法

研究の一部業務を外部委託する。
（1）患者割付、データ入力、統計解析、モニタリング

・委託先：東北大学病院臨床研究データセンター
（2）調整事務局（一部）
・委託先：シミックヘルスケア・インスティテュート株式会社　SSIカンパニー
監督方法
提供元は、匿名化されている情報のみを提供し、個人識別符号を含む（研究対象者コードリストを含む）データは提供しない。情報提供に伴い、提供に関する記録を作成・保存する。保管期間は、提供元（研究事務局）は論文化から5年間、提供先は臨床研究法に定められた期間とする。

18.4. 個人情報等の取扱い

18.4.1. 個人情報の利用目的

研究の正しい結果を得るために、治療中だけではなく治療終了後も長期間にわたり研究対象者個人を特定して調査を行うこと、取得した情報を適切に管理することを目的として個人情報を利用する。

18.4.2. 利用方法（匿名化の方法）

（1）個人情報等の有無について

種類		定義	具体例	有無
個人情報		①情報単体で特定の個人を識別することができるもの	氏名・顔画像等	■有<br>□無
		②他の情報と照合することによって特定の個人を識別することができるもの	対応表によって特定の個人を識別することができる他の情報と照合できるもの	
		③個人識別符号が含まれるもの	ゲノムデータ　等	□有<br>（具体的に：　　　）<br>■無
	要配慮個人情報	病歴、社会的身分、人種、信条、犯罪の経歴、犯罪により害を被った事実その他本人に対する不当な差別、偏見その他の不利益が生じないようにその取扱いに特に配慮を要する記述等が含まれる個人情報	診療録、レセプト、健診の結果、一部のゲノム情報※等	■有<br>（具体的に：診療録）<br>□無

※個人識別符号に該当するゲノムデータに単一遺伝子疾患、疾患へのかかりやすさ、治療薬の選択に関するものなどの解釈を付加し、医学的意味合いを持った「ゲノム情報」は、要配慮個人情報に該当する場合がある。

（2）匿名化の有無

■匿名化する（(3) へ）

☐匿名化しない（理由：　　　　　　　　　）
☐その他（具体的に：　例：行政機関/独立行政法人等個人情報保護法の規定の適用を受ける非識別加工情報とする。）

(3)匿名化の種類及び方法

■1）匿名化されている。（特定の個人を識別することができる対応表を本学で作成しているものに限る）

方法：研究対象者のデータや検体から氏名等の特定の個人を識別することができることとなる記述等を削り、代わりに新しく符号又は番号をつけて匿名化を行う研究対象者とこの符号（番号）を結びつける対応表を各共同研究機関で作成してその機関で保管作成し、個人情報管理者は外部に漏れないように厳重に保管する。

18.4.3. 安全管理責任体制（個人情報の安全管理措置）

共同研究を行う医療機関ごとにCRFおよび対応表を作成する。CRF原本、対応表は東北大学病院臨床試験データセンターで鍵付きの保管庫に施錠の上保管する。CRFの管理も同様である。

18.5.　研究対象者等、その関係者からの相談等への対応

本研究に関する場合
研究代表医師：高山 真
東北大学病院　総合地域医療教育支援部・漢方内科　准教授
〒980-8574　宮城県仙台市青葉区星陵町1-1
TEL: 022-728-3036　E-mail : takayama@med.tohoku.ac.jp

本研究に関連する医師や医療スタッフ以外の者に相談したいことがある場合
東北大学臨床研究審査委員会事務局 相談窓口
TEL：022-718-0461（受付時間：平日9時～17時）
E-mail: office@nrs.hosp.tohoku.ac.jp

18.6.研究計画書の変更

研究計画書を変更する場合、研究代表医師は、認定臨床研究審査委員会の審査を経て研究機関の長の承認を得る。

研究計画書内容の変更を、改正・改訂の2種類に分けて取扱う。その他、研究計画書の変更に該当しない補足説明の追加をメモランダムとして区別する。

(1) 改正（Amendment）

研究対象者の危険を増大させる可能性のある、または主要評価項目に影響を及ぼす研究計画書の変更。各研究機関の承認を要する。以下の場合が該当する。

①研究対象者に対する負担を増大させる変更（採血、検査等の侵襲の増加）
②重篤な副作用情報による変更（除外基準等）
③有効性・安全性の評価方法の変更
④症例数の変更

⑵ 改訂（Revision）

研究対象者の危険を増大させる可能性がなく、かつ主要評価項目に影響を及ぼさない研究計画書の変更。各研究機関の承認を要する。以下の場合が該当する。

①研究対象者に対する負担を増大させない変更（検査時期の変更）

②研究期間の変更

③研究者の変更

⑶ メモランダム／覚え書き（Memorandum）

研究計画書内容の変更ではなく、文面の解釈上のバラツキを減らす、特に注意を喚起する等の目的で、研究責任医師から研究関係者に配布する研究計画書の補足説明。

18.7. 総括報告書

研究代表医師は、総括報告書を作成し、認定臨床研究審査委員会に提出する。

研究代表医師は、認定臨床研究審査委員会承認後、総括報告書の概要（規則第 24 条 別紙様式 1 終了届出書）を jRCT に公開するとともに、厚生労働大臣に提出する。

承認された総括報告書は、各施設の研究責任医師を通じて各実施医療機関の管理者に提出する。

19. 引用文献

文献１　国家卫生健康委员会.新型冠状病毒感染的肺炎诊疗方案 (試行第七版)的通知

文献２　王饶琼，杨思进，谢春光，沈其霖，李敏清，雷枭，李继科，黄梅. 清肺排毒汤治疗新型冠状病毒肺炎的临床疗效观察. 中药药理与临床, 2020 (中国語、Online first)

文献３　中国における COVID-19 に対する清肺排毒湯の報告, 有田龍太郎 髙山 真 石沢 興太 石井正, 日本感染症学会 2020.4.21, http://www.kansensho.or.jp/uploads/files/news/gakkai/covid19_kiko_0421.pdf

改定履歴（使用時は本ページを削除）

版番号	作成・改定日	改定理由／内容
第 1.0 版	2020 年 5 月 1 日	新規制定
第 1.1 版	2020 年 7 月 3 日	一部修正
